# Long-Term Effect of GPi-DBS in a Patient With Generalized Dystonia Due to GLUT1 Deficiency Syndrome

**DOI:** 10.3389/fneur.2018.00381

**Published:** 2018-05-30

**Authors:** Idil Hanci, Christoph Kamm, Marlieke Scholten, Lorenzo P. Roncoroni, Yvonne Weber, Rejko Krüger, Christian Plewnia, Alireza Gharabaghi, Daniel Weiss

**Affiliations:** ^1^Department of Neurodegenerative Diseases, Hertie Institute for Clinical Brain Research, University of Tübingen, Tübingen, Germany; ^2^German Centre of Neurodegenerative Diseases, University of Tübingen, Tübingen, Germany; ^3^Centre for Integrative Neuroscience, University of Tübingen, Tübingen, Germany; ^4^Department of Neurology, University of Rostock, Rostock, Germany; ^5^Graduate School of Neural and Behavioral Sciences, International Max Planck Research School, University of Tübingen, Tübingen, Germany; ^6^Department of Neurology and Epileptology, Hertie Institute for Clinical Brain Research, University of Tübingen, Tübingen, Germany; ^7^Clinical and Experimental Neuroscience, Luxembourg Centre for Systems Biomedicine, Centre Hospitalier de Luxembourg, University of Luxembourg, Luxembourg, Luxembourg; ^8^Department of Psychiatry and Psychotherapy, Neurophysiology and Interventional Neuropsychiatry, University of Tübingen, Tübingen, Germany; ^9^Division of Functional and Restorative Neurosurgery, Department of Neurosurgery, University of Tübingen, Tübingen, Germany

**Keywords:** glucose transporter type 1 (GLUT1), glucose transporter type-1 deficiency syndrome (GLUT1-DS), deep brain stimulation (DBS), globus pallidus internus (GPi), dystonia

## Abstract

Treatment outcomes from pallidal deep brain stimulation are highly heterogeneous reflecting the phenotypic and etiologic spectrum of dystonia. Treatment stratification to neurostimulation therapy primarily relies on the phenotypic motor presentation; however, etiology including genetic factors are increasingly recognized as modifiers of treatment outcomes. Here, we describe a 53 year-old female patient with a progressive generalized dystonia since age 25. The patient underwent deep brain stimulation of the globus pallidus internus (GPi-DBS) at age 44. Since the clinical phenotype included mobile choreo-dystonic features, we expected favorable therapeutic outcome from GPi-DBS. Although mobile dystonia components were slightly improved in the long-term outcome from GPi-DBS the overall therapeutic response 9 years from implantation was limited when comparing “stimulation off” and “stimulation on” despite of proper electrode localization and sufficient stimulation programming. In order to further understand the reason for this limited motor symptom response, we aimed to clarify the etiology of generalized dystonia in this patient. Genetic testing identified a novel heterozygous pathogenic *SLC2A1* mutation as cause of glucose transporter type 1 deficiency syndrome (GLUT1-DS). This case report presents the first outcome of GPi-DBS in a patient with GLUT1-DS, and suggests that genotype relations may increasingly complement phenotype-based therapy stratification of GPi-DBS in dystonia.

## Case presentation

A 44-year-old female patient with generalized dystonia including mobile components underwent bilateral deep brain stimulation of the globus pallidus internus (GPi-DBS; electrode 3387; Medtronic, Minneapolis, MN, USA). When the patient showed limited response to GPi-DBS years from DBS surgery, we performed a detailed diagnostic work-up. This revealed a pathogenic mutation of the glucose transporter 1 deficiency syndrome (GLUT1-DS).

The perinatal history was unremarkable, no medical records on motor and speech development was available. She presented with first symptoms in terms of learning disability at age six. Moreover, complex partial seizures occurred in childhood and were successfully treated with carbamazepine. Remission from seizures was reported at age 18. At age 25, both dystonic and choreatic movements as well as action and postural tremor of the right hand appeared. At age 27, cervical dystonia was documented. Finally, at age 37, progression of dystonic symptoms was reported with dystonia affecting the lower limbs resulting in dystonic gait impairment. Family history of the non-consanguineous parents and three siblings was unremarkable with respect to dystonia or other neurologic diseases.

Pharmacological treatment did not improve dystonia (tiapride up to 600 mg/d, trihexyphenidyl 10 mg/d, L-Dopa 500 mg/d, or baclofen 15 mg/d). The patient did not tolerate tetrabenazine due to depressive symptoms. No premorbid exposure to neuroleptics was documented.

In the preoperative state, generalized dystonia including both tonic and mobile choreo-dystonic movement components was the leading motor presentation that prompted DBS therapy (Supplemental Video 1). Severity of dystonia was rated based on a preoperative video including the Global Dystonia Rating Scale (GDS), the Fahn Marsden Rating Scale (F-M) and the Unified Dystonia Rating Scale (UDRS). No quantitative assessment of dystonia motor scores is available from the first years after DBS implantation. However, narrative information from the patient records (neurologist perspective) supported a stun effect 1 week after DBS with improvements of cervical dystonia and choreo-dystonic movements of the right arm. This early effect was reported to be attenuated at 3 months postoperatively but still superior to the preoperative state. At 15 months from surgery, improvement of cervical dystonia compared to the preoperative situation was reported. Then, the patient did not present again for detailed clinical inpatient assessments, except IPG replacements and battery checks for several years.

We were able to perform an in-depth characterization of the formal GPi-DBS effect at 9 years from surgery when the patient was referred to the inpatient neurology ward. At this time, generalized dystonia still was the leading motor presentation, including cervical dystonia and dystonia of both upper extremities and the right leg. In addition, moderate postural and action tremor of the right arm and mild signs of cerebellar dysfunction in terms of a slightly dysmetric finger-to-finger test, intention tremor, and hypo metric saccades of horizontal eye movements were present (detailed clinical examination in Supplemental Material). We obtained “on stimulation” scores (stimulation parameters: left GPi: 1-C+, 3.0 V, 120 μs, 150 Hz; right GPi: 4-C+, 5.5 V, 180 μs, 150 Hz) and “off stimulation” scores, the latter after having discontinued GPi-DBS for 3 days under inpatient clinical observation. Both postoperative conditions were videotaped and scores were obtained by a rater blinded to the therapeutic condition (excerpts of these videos are provided as Supplemental Video 2 (“stimulation off”) and Supplemental Video 3 (“stimulation on”) for comparison with the preoperative state).

Preoperative scores were as follows: GDS 74/140, UDRS 71/224, F-M 56.5/120. At this time, dystonia was most pronounced in the lower face with perioral and periorbicular cramping, cervical dystonia, dysarthria without spasmodic dysphonia, and dystonia of the right arm. In particular, dystonia of the right distal arm and cervical dystonia included pronounced mobile components. Nine years from DBS implantation, two dystonia scores showed mild improvements when comparing “stimulation off” and “stimulation on,” i.e., GDS (off: 72/140; on: 65/140) and UDRS (off: 89/224; on: 64.5/224). The F-M was similar across conditions (off: 56.5/120; on: 58/120). After discontinuing GPi-DBS, no rebound of dystonia was observed. However, in both “stimulation on” and “stimulation off” conditions, cervical dystonia and dystonia of the right upper extremity was less mobile as compared to the preoperative state. Instead, the tonic components of cervical and upper extremity dystonia were more pronounced in the postoperative long-term follow-up as compared to the preoperative state. Moreover, there seemed to be more axial dystonia in both “stimulation off” and “stimulation on” as compared to the preoperative stage including an intermediate increase in torsion of the trunk, pelvis tilt during gait, and slower and bradykinetic gait patterns.

Owing to the mild but limited clinical motor effect of GPi-DBS, we reassessed the localization of active electrode contacts, which were in accordance with standards described elsewhere (1, 2) (mid-commissural point based coordinates of active contacts: left: 21.1 mm lateral, 1.3 mm anterior, 2.9 mm inferior; right: 19.3 mm lateral, 0.3 mm anterior, 1.4 mm inferior). Moreover, we ensured that DBS parameters were set according to the current reprogramming standard that considers high intensity stimulation generally on the caudal electrode contacts in localizations as detailed above. As such, we verified from the patient records that the two lowermost contacts of both electrodes were repeatedly adjusted for chronic stimulation and maintained for prolonged time intervals of months and years (the parameter space included left Gpi: contacts 0 or 1; single monopolar or bipolar configurations; amplitudes: 2.5–4.0 V (range), pulse widths: 120–210 μs (range); right Gpi: contacts 4 or 5, single monopolar or bipolar configuration; amplitudes: 2.5–5.5 V (range); pulse widths: 120–210 μs (range); frequencies: 130–200 Hz (range). Electrode impedances were normal and there was no indication for malfunction of the stimulators.

We also reasoned whether the more bradykinetic gait at the postoperative 9-year follow-up would reflect an adverse effect of GPi-DBS as described previously (3). However, gait kinematic temporal measures showed larger stride length and faster stride velocity in “stimulation on” as compared to “stimulation off,” and this argues for a slight beneficial instead of an adverse effect (Table 1).

**Table 1:** Gait characteristics.

	**Postoperativ “stimulation on”**	**Postoperativ “stimulation off”**
Mean stride length L (%stature)	39.37	23.05
Mean stride length R (%stature)	36.13	20.85
Mean stride velocity L (%stature/s)	25.32	15.60
Mean stride velocity R (%stature/s)	23.31	14.09
Mean gait cycle time (s)	1.56	1.52

*The patient performed a seven-meter walking test (back and forth including turning). Sensors comprising tri-axial accelerometer, gyroscope, and magnetometer were attached to both ankles lumbar position (Opal, APMD Inc, Portland, OR, USA). Kinematic gait measures were computed with Mobility Lab (APMD Inc, Portland, OR, USA) (4)*.

To further investigate possible reasons for the mild but limited therapeutic response, we aimed to clarify the etiologic basis of generalized dystonia. Therefore, we initiated molecular genetic testing with next generation sequencing and employed a gene panel for generalized dystonia. This revealed a heterozygous *SLC2A1* deletion (c.972 + 1delG) (deletion, affecting splice site) indicative for GLUT1-DS, which is pathogenic owing to *in silico* predictions (www.mutationtaster.org). Moreover, other pathogenic mutations were described previously in c.972 position (5). We excluded other dystonia-related mutations (Supplemental Material). After diagnosing GLUT1-DS, we referred the patient to modified Atkin's diet which was discontinued by the patient after 8 weeks. Moreover, the patient refused lumbar puncture and CSF glucose analysis.

The patient and the legal representative provided written informed consent and authorization of video publications for this case report.

## Background

GLUT1-DS (OMIM #606777) arises from impaired glucose transport into the brain because of *SLC2A1* mutations. The syndrome was first described in 1991 (6), and may present considerable phenotypic variability potentially including infantile seizures, developmental delay, cognitive impairment, microcephaly and various movement disorders (7). Most common movement disorders include dystonia, chorea and myoclonus (8) as well as exercise-induced dyskinesias (9). Clinical variability depends on the type of mutation in *SLC2A1* gene (10). Ketogenic diet and modified Atkins diet show variable efficacy on clinical symptoms (11, 12) with better effects on frequency of seizures (13) and less pronounced effect on movement disorder symptoms (14). So far, no cases with GLUT1-DS and GPi-DBS for dystonic symptoms were reported, however, from a phenotype perspective, GPi-DBS indication was plausible at the time of implantation, since the patient had generalized dystonia including mobile choreo-dystonic movement components.

## Discussion

We consider the novel heterozygous *SLC2A1* variant (c.972 + 1delG) as pathogenic mutation, since both clinical features and disease course are consistent with previously described phenotypes of GLUT1-DS. Moreover, mutations in similar regions of the *SLC2A1* gene were reported in other patients with classical GLUT1-DS (7, 15, 16), including deletions of the c.972 position (5), which is located at splice site. Since there was no evidence for paroxysmal dyskinesias in our patient - as is frequently observed in patients with GLUT1-DS (9, 17) - GLUT1-DS was initially not considered as differential diagnosis when referring to GPi-DBS. Therefore, molecular genetic panel testing including the *SLC2A1* gene was initiated with temporal delay from GPi-DBS owing to dystonia panel analysis.

There was narrative information on a mild improvement of right arm dystonia and torticollis early after Gpi-DBS from the patient records. In the long-term, however, postoperative scores in both “stimulation off” and “stimulation on” were similar to the preoperative state. This includes the lack of dystonia rebound in the “stimulation off” state which may point to limited efficacy of GPi-DBS in this patient. Together the findings support that dystonia was less mobile at follow-up compared to the preoperative state; vice versa there seemed to be more axial involvement. Together, this may point to a mild improvement of mobile components, and mild axial disease progression over 9 years resulting in similar pre- and postoperative global motor scores.

Closer follow-up assessments after surgery would have been of value to further substantiate this impression, however, are not available, since the patient did not wish to conduct the annual inpatient control visits, that are generally standard at our institute as standard operating procedure (including quantitative follow-up scoring). Nevertheless, there is indication for a mild but limited DBS effect. For comparison, motor symptoms generally recur within minutes in effective GPi-DBS when DBS is intercepted (18). Moreover, after discontinuing GPi-DBS for 72 h there was no clear prolonged washout of a potential GPi-DBS effect. Interestingly, however, the mobile dystonia component was still improved in both postoperative conditions as compared to the preoperative state, which might relate either to neuroplastic effects of GPi-DBS (19) or to phenotype alterations over the long-standing and progressive disease course.

Together, we initially expected more favorable outcome owing to generalized dystonia with mobile components as leading motor presentation. However, as in other hereditary dystonia syndromes, genotype relations may affect therapeutic outcomes with favorable clinical response such as in *DYT1* mutations or, vice-versa, near-to-absent response as for example in rapid-onset dystonia parkinsonism (*DYT12*). In this aspect, we provide first indication that *SLC2A1* mutations might associate with a mild but limited therapeutic effect. Future observations are needed to further substantiate this first observation.

## Conclusion

In summary, we report the first patient with GLUT1-DS due to a novel heterozygous *SLC2A1* mutation treated with GPi-DBS. Generalized dystonia including mobile components was the leading presentation at the preoperative state. There was indication for initial mild improvement of right arm dystonia and torticollis, however, in the long-term the therapeutic effect was limited. This case report lends further support to the relevance of molecular genetic testing of patients with generalized dystonia in attempt to stratify DBS outcomes. Molecular genetic diagnostics may increasingly add to understand heterogeneity in DBS outcomes in generalized dystonia (20, 21).

## Ethics statement

We confirm that the approval of an institutional review board was not required for this work and that we have read the Journal's position on issues involved in ethical publication and affirm that this work is consistent with those guidelines.

## Author contributions

IH, AG, and DW: design or conceptualization of the study; IH, CK, MS, LR, and DW: analysis or interpretation of the data; IH, CK, MS, LR, YW, RK, CP, AG, and DW: drafting or revising the manuscript for intellectual content.

### Conflict of interest statement

The authors declare that the research was conducted in the absence of any commercial or financial relationships that could be construed as a potential conflict of interest.
