# Supplementary material for: Long-Term Effect of GPi-DBS in a Patient With Generalized Dystonia Due to GLUT1 Deficiency Syndrome
Source: Front Neurol. 2018 May 30;9:381. doi: 10.3389/fneur.2018.00381 (PMC5988881; doi:10.3389/fneur.2018.00381)
Supplement: Supplementary file 4 [file Data_Sheet_1.DOCX]

**Clinical examination**

The dystonia presented with shoulder adduction, elbow flexion, palmar flexion of wrist, flexion of all right hand fingers of right upper extremity; wrist dorsiflexion, abduction of the left hand fingers of left upper extremity; internal rotation of the right thigh, supination of the right foot. Cervical dystonia included left laterocollis 10° and laterocaput 45°, right torticollis 30°, antecollis 15° and dystonic elevation of the left shoulder. Moderate postural and action tremor of the right arm.

In addition, there were mild signs of cerebellar dysfunction with intention tremor of the right hand, broad-based dysrhythmic gait, dysmetric finger-to-finger test and hypo metric saccades of horizontal eye movements.

**Excluded dystonia-related mutations**

*ANO3, ARSA, ATM, ATP1A3, ATP7A, ATP7B, AUH, BCAP31, CACNA1B, CIZ1, CLN3, COL6A3, COX20, CSF1R, CYP27A1, FTL, GCDH, GCH1, GNAL, HEPACAM, HEXA, KCNMA1, KCTD17, KIF1C, MECP2, MLC1, NPC1, NPC2, PANK2, PARK2, PLA2G6, PNKD, PRKRA, PRRT2, SGCE, SLC25A15, SLC30A10, SMPD1, SPR, TAF1, TH, THAP1, TIMM8A, TOR1A, TUBB4A, VPS13*A
